# DARS-AS1: A Vital Oncogenic LncRNA Regulator with Potential for Cancer Prognosis and Therapy

**DOI:** 10.7150/ijms.90611

**Published:** 2024-01-20

**Authors:** Jian Shu, Kejiang Xia, Hongliang Luo, Yang Wang

**Affiliations:** 1 Department of Gastrointestinal Surgery, The Second Affiliated Hospital, Jiangxi Medical College, Nanchang University, Nanchang 330008, Jiangxi, China.; 2 Department of Spleen and Stomach Diseases, Jiujiang Hospital of Traditional Chinese Medicine, Jiujiang 332000, Jiangxi, China.; 3 Department of Neurosurgery, Yingtan People's Hospital, Yingtan 335000, Jiangxi, China.

**Keywords:** LncRNA, DARS-AS1, Tumor biomarker, Biological Functions, Regulatory mechanism

## Abstract

DARS-AS1, short for Aspartyl-tRNA synthetase antisense RNA 1, has emerged as a pivotal player in cancers. Upregulation of this lncRNA is a recurrent phenomenon observed across various cancer types, where it predominantly assumes oncogenic roles, exerting influence on multiple facets of tumor cell biology. This aberrant expression of DARS-AS1 has triggered extensive research investigations, aiming to unravel its roles and clinical values in cancer. In this review, we elucidate the significant correlation between dysregulated DARS-AS1 expression and adverse survival prognoses in cancer patients, drawing from existing literature and pan-cancer analyses from The Cancer Genome Atlas (TCGA). Additionally, we provide comprehensive insights into the diverse functions of DARS-AS1 in various cancers. Our review encompasses the elucidation of the molecular mechanisms, ceRNA networks, functional mediators, and signaling pathways, as well as its involvement in therapy resistance, coupled with the latest advancements in DARS-AS1-related cancer research. These recent updates enrich our comprehensive comprehension of the pivotal role played by DARS-AS1 in cancer, thereby paving the way for future applications of DARS-AS1-targeted strategies in tumor prognosis evaluation and therapeutic interventions. This review furnishes valuable insights to advance the ongoing efforts in combating cancer effectively.

## Introduction

Initially, long non-coding RNAs (lncRNAs) were considered as transcriptional noise or "junk RNA" since they lacked the functionality of encoding proteins 1-3. However, as scientific research has advanced, it has become evident that lncRNAs play crucial roles in gene regulation 4-8, involving processes such as transcription, splicing, translation, and chromatin structure modifications. LncRNAs have been found to play key roles in various diseases 9-13, particularly in cancer 14-16. Moreover, lncRNAs have the potential to serve as valuable biomarkers for progression and prognosis assessment, as well as promising therapeutic targets 17-22. With the development and widespread use of high-throughput sequencing technologies, an increasing number of lncRNAs have been discovered 23-26.

LncRNAs are classified into several categories, such as antisense, sense, intronic, and intergenic, each possessing unique characteristics 27. Focusing on antisense lncRNAs, these transcripts are uniquely synthesized from the opposite DNA strand of genes that may encode proteins or serve non-coding functions, leading to their critical role in the onset and development of tumors, which has recently garnered attention 28-31. At the molecular level, they execute regulatory functions through a series of mechanisms, encompassing epigenetic modification, transcriptional control, post-transcriptional regulation, and impacts on translation 16, 28, 32. Additionally, due to their nucleotide sequence complementarity, antisense lncRNAs possess a distinctive regulatory capability, specifically targeting and interacting with their corresponding sense genes 28, further highlighting the complexity of their involvement in cellular and pathological processes, particularly within the realm of cancer.

**Figure 1:**
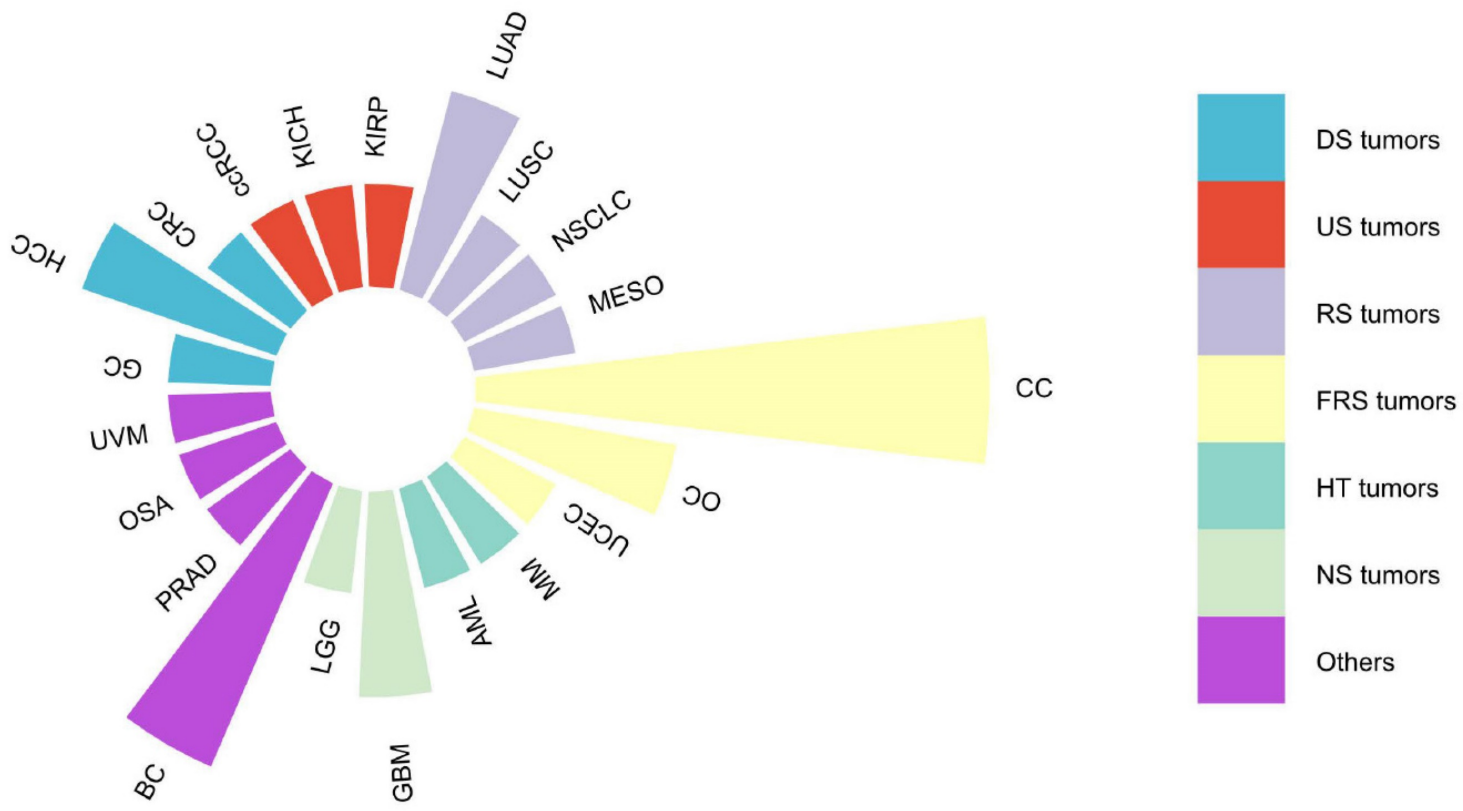
Malignancies associated with lncRNA DARS-AS1 in existing literature studies. This figure summarizes the association between various malignancies and lncRNA DARS-AS1 reported in the existing literature. One publication studied the link between lncRNA DARS-AS1 and GC, CRC, ccRCC, KICH, KIRP, LUSC, NSCLC, MESO, UCEC, MM, AML, LGG, PRAD, OSA, and UVM. Two publications studied the link between lncRNA DARS-AS1 and HCC, LUAD, OC, and GBM. Three publications studied the role of lncRNA DARS-AS1 in BC. And five publications studied the effects of lncRNA DARS-AS1 in CC. **Abbreviations:** CC: Cervical cancer, OC: Ovarian cancer, UCEC: Uterine corpus endometrial carcinoma, MM: Myeloma, AML: Acute myeloid leukemia, GBM: Glioblastoma, LGG: Lower grade glioma, BC: Breast cancer, PRAD: Prostate adenocarcinoma, OSA: Osteosarcoma, UVM: Uveal melanoma, GC: Gastric cancer, HCC: Hepatocellular carcinoma, CRC: Colorectal cancer, ccRCC: Clear cell renal cell carcinoma, KICH: Kidney chromophobe, KIRP: Kidney renal papillary cell carcinoma, LUAD: Lung adenocarcinoma, LUSC: Lung squamous cell carcinoma, NSCLC: Non-small cell lung cancer, MESO: Mesothelioma. DS tumors: Digestive system tumors, US tumors: Urinary system tumors, RS tumors: Respiratory system tumors, FRS tumors: Female reproductive system tumors, HT tumors: Hematological tumors, NS tumors: Neurological system tumors.

DARS-AS1, or Aspartyl-tRNA synthetase antisense RNA 1, serves as a prime example of an lncRNA, situated on chromosome 2 at q21.3 of the human genome. Spanning 22,367 nucleotides and comprising five exons (source: https://www.ncbi.nlm.nih.gov/gene/101928243), DARS-AS1 gives rise to a remarkable 34 splice variants, ranging in size from 311 base pairs for DARS1-AS1-228 to 1634 base pairs for DARS1-AS1-233(source: https://www.ensembl.org/Homo_sapiens/Gene/Summary?g = ENSG00000231890; r = 2:135985124-136022593). As an antisense lncRNA, DARS-AS1 has emerged as a recognized oncogenic entity, implicated in a wide array of cancer types as supported by extensive research publications 33-51, as depicted in **Figure 1**. These encompass cancers originating from various organ systems, including the digestive, urinary, respiratory, female reproductive, hematological, and neurological systems, among others. Notably, elevated DARS-AS1 expression has been linked to unfavorable clinicopathological characteristics and diminished overall survival outcomes. DARS-AS1 plays a pivotal part in tumor-related processes such as cell proliferation, migration, invasion, and autophagy, and potentially influences the sensitivity of tumor cells to radiotherapy or chemotherapy. These effects are mainly mediated through a complex ceRNA network and signaling pathways, which regulate tumorigenesis and disease progression. Here, we provide a concise review based on existing literature and The Cancer Genome Atlas (TCGA) analysis, mainly around examining the clinical significance of DARS-AS1, its impact on regulating cellular processes and related mechanisms, and its potential as a promising therapeutic target.

### Expression of DARS-AS1 in tumor tissues

DARS-AS1, an emerging tumor marker, exhibits significant upregulation in a diverse array of cancer types based on research involving in-house tissue specimens and analyses of TCGA data (**Table 1**). These cancer types span various bodily systems, including the neurological system (such as brain lower-grade glioma and glioblastoma), the respiratory system (including non-small cell lung cancer, and mesothelioma), the digestive system (comprising gastric cancer, liver cancer, and colorectal cancer), the urinary system (featuring renal cell carcinoma), the female reproductive system (encompassing cervical and ovarian cancers), and the hematological system (including myeloma and acute myeloid leukemia). Additionally, DARS-AS1 demonstrates upregulation in other tumor types, such as bladder cancer, prostate adenocarcinoma, uveal melanoma, breast cancer, and osteosarcoma. These findings suggest that DARS-AS1 may hold significance as a potential biomarker or therapeutic target across a wide spectrum of cancers, warranting further investigation into its specific roles and clinical applications.

### LncRNA DARS-AS1 is associated with tumor-related clinical features

Several investigations have explored the relationship between DARS-AS1 expression and clinicopathological characteristics across a spectrum of five tumor types (**Table 1**). In gastric cancer 51, DARS-AS1 exhibits a significant positive correlation with T category, N category, TNM stage. In hepatocellular carcinoma 35, overexpression DARS-AS1 indicates larger tumor size and advanced TNM stage. In clear cell renal cell carcinoma 36 and triple-negative breast cancer 40, high expression of DARS-AS1 is indicative of higher clinical-stage. In acute myeloid leukemia 34, patients with high DARS-AS1 expression exhibit a significantly higher leukocyte count in peripheral blood, while their hemoglobin levels and platelet counts are notably lower compared to those with low DARS-AS1 expression.

### LncRNA DARS-AS1 is a valuable prognostic marker

LncRNA DARS-AS1 serves as a valuable prognostic indicator. The abnormal expression of DARS-AS1 has been closely associated with overall survival (OS) in cancer patients. As summarized in **Table 1**, drawing from both the in-house survival data and TCGA analyses conducted in these studies for OS, high levels of DARS-AS1 expression are predictive of poorer OS in a range of cancer types, including gastric cancer, hepatocellular carcinoma, lung adenocarcinoma, triple-negative breast cancer, cervical cancer, acute myeloid leukemia, UVM, KICH, KIRP, MESO, GBM, and LGG.

In addition to the effect on OS, we also performed a comprehensive assessment of the prognostic relevance of DARS-AS1 in a pan-cancer analysis using TCGA, considering disease-specific survival (DSS), disease-free interval (DFI) and progression-free interval (PFI).

**Figure 2:**
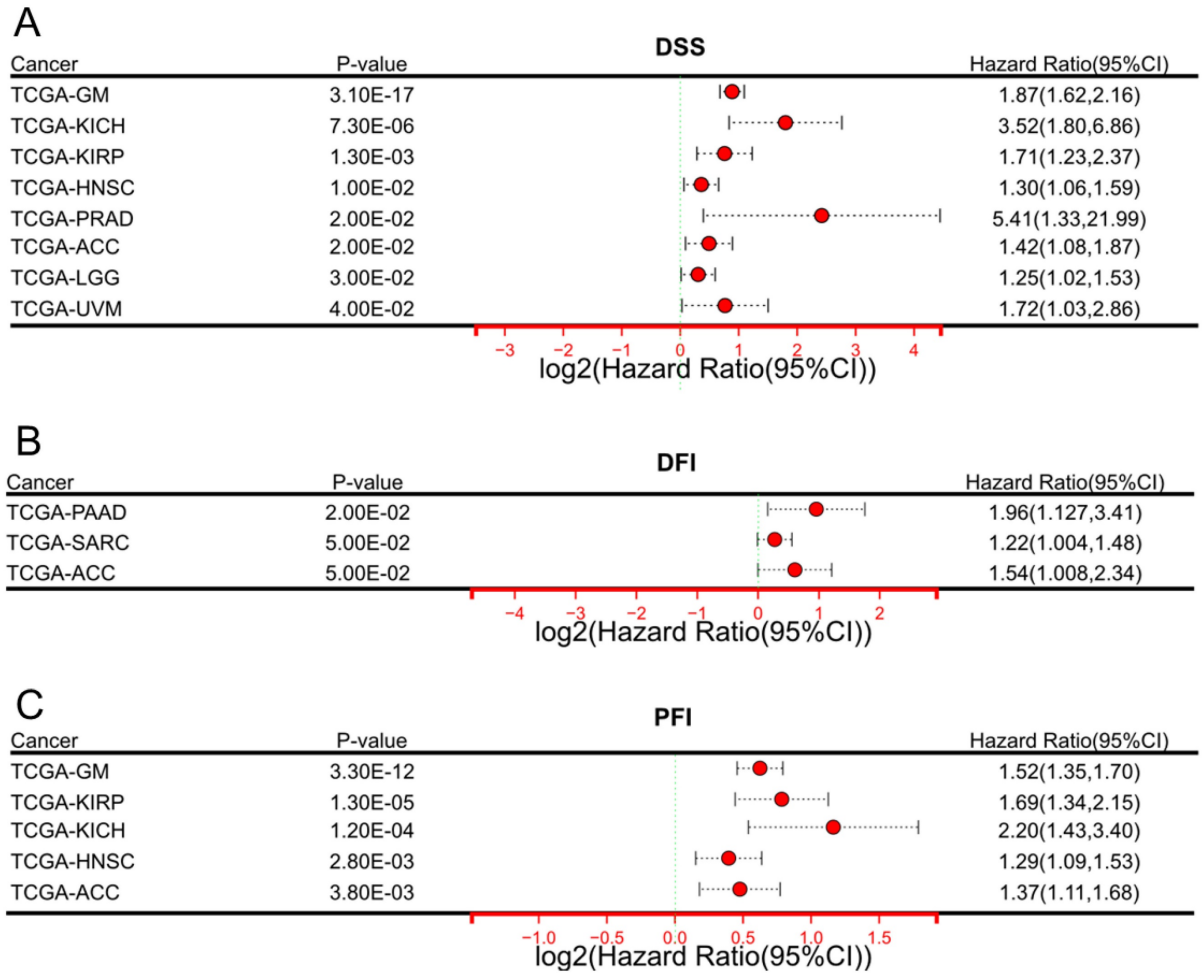
Significant prognostic significance of lncRNA DARS-AS1 for DSS/DFI/PFI in pan-cancer analysis using TCGA. (A) High expression levels of lncRNA DARS-AS1 are associated with poorer DSS in GM, KICH, KIRP, HNSC, PRAD, ACC, LGG, and UVM. (B) Elevated expression of lncRNA DARS-AS1 is linked to inferior DFI specifically in PRAD, SARC, and ACC. (C) High expression levels of lncRNA DARS-AS1 are indicative of poorer PFI in GM, KIRP, KICH, HNSC, and ACC. **Abbreviations:** DSS: Disease-specific survival; DFI: Disease-free interval; PFI: Progression-free interval; GM: Glioma; KICH: Kidney chromophobe; KIRP: Kidney renal papillary cell carcinoma; HNSC: Head and neck squamous cell carcinoma; PRAD: Prostate adenocarcinoma; ACC: Adrenocortical carcinoma; LGG: Lower grade glioma; UVM: Uveal melanoma; SARC: Sarcoma.

In terms of DSS (**Figure 2A**), elevated expression of DARS-AS1 indicated unfavorable prognosis in eight tumor types, namely TCGA-GM (HR = 1.87(1.62,2.16), TCGA-KICH (HR=3.52(1.80,6.86), TCGA-KIRP (HR=1.71(1.23,2.37), TCGA-HNSC (HR = 1.30(1.06,1.59), TCGA-PRAD (HR=5.41(1.33,21.99), TCGA-ACC (HR=1.42(1.08,1.87),TCGA-LGG (HR = 1.25(1.02,1.53), and TCGA-UVM (HR=1.72(1.03,2.86), where high expression was indicative of poorer outcomes.

In terms of DFI (**Figure 2B**), increased DARS-AS1 expression was linked to a poor prognosis in three tumor types, TCGA-PAAD (HR = 1.96(1.127,3.41), TCGA-SARC (HR = 1.22(1.004,1.48) and TCGA-ACC (HR = 1.54(1.008,2.34) where high expression was associated with worse outcomes.

Regarding PFI (**Figure 2C**), it was observed that high expression of DARS-AS1 led to poorer prognosis in five tumor types, namely TCGA-GM (HR = 1.52(1.35,1.70), TCGA-KIRP (HR = 1.69(1.34,2.15), TCGA-KICH (HR = 2.20(1.43,3.40), TCGA-HNSC (HR = 1.29(1.09,1.53), and TCGA-ACC (HR = 1.37(1.11,1.68), highlighting the adverse impact of elevated DARS-AS1 expression on prognosis.

### Functions of DARS-AS1 in human tumors

**Figure 3:**
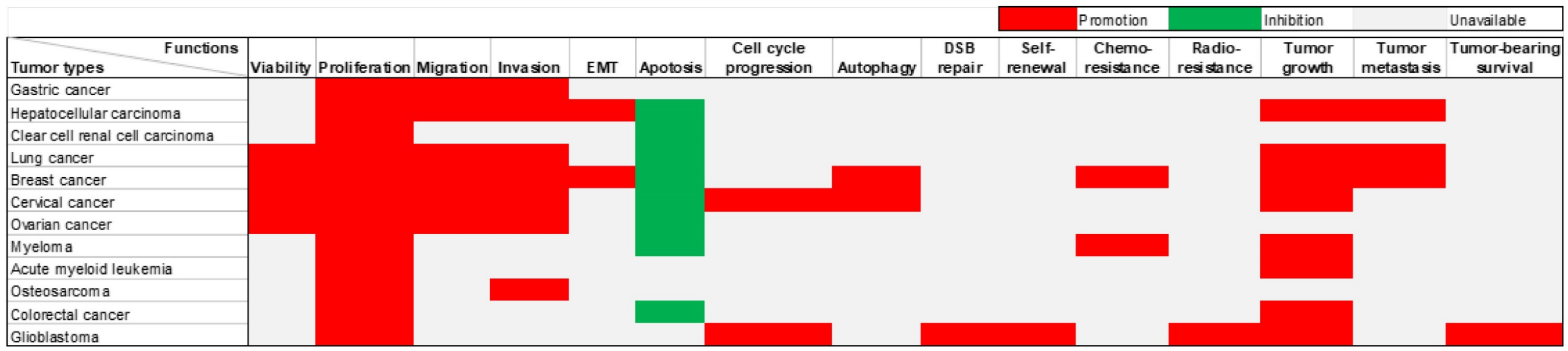
Diverse roles of lncRNA DARS-AS1 in twelve distinct human tumors through *in vitro* and/or *in vivo* experiments. LncRNA DARS-AS1 exhibits oncogenic effects in gastric, liver, kidney, lung, breast, cervical, ovarian, and colorectal cancers, as well as myeloma, acute myeloid leukemia, osteosarcoma, and glioblastoma. **Abbreviations:** EMT: Epithelial-mesenchymal transition; DSB repair: double-strand break repair.

Extensive research has been conducted on the role of DARS-AS1 in twelve different types of tumors, utilizing *in vivo* and/or *in vitro* experiments (**Table 2**). DARS-AS1 expression has been consistently observed to be up-regulated in numerous tumor cell lines. The subcellular localization of this lncRNA was reported within the cytoplasm in six different types of tumor cells, including clear cell renal cell carcinoma, cervical cancer, acute myeloid leukemia, colorectal cancer, and glioblastoma cells. DARS-AS1 plays a pivotal oncogenic role in tumor development, impacting a series of biological processes (**Figure 3**). It promotes epithelial-mesenchymal transition (EMT), augments cell proliferation, enhances cell viability, facilitates migration and invasion, induces autophagy, contributes to therapy resistance, facilitates tumor growth, and metastasis. Conversely, DARS-AS1 inhibits apoptosis and hinders cell cycle arrest in tumor cells. These findings emphasize the profound importance of DARS-AS1 as a critical oncogenic regulator influencing various aspects of tumor progression.

### ceRNA network involving lncRNA DARS-AS1 in tumor progression

In recent years, there has been a notable surge in the exploration of competing endogenous RNAs (ceRNAs) networks 52-56. These ceRNAs function as defensive shields, safeguarding mRNAs from the inhibitory actions of miRNAs 57. Within the cancer research, the ceRNA regulatory network orchestrated by lncRNAs plays a pivotal and indispensable role 58-62.

**Figure 4:**
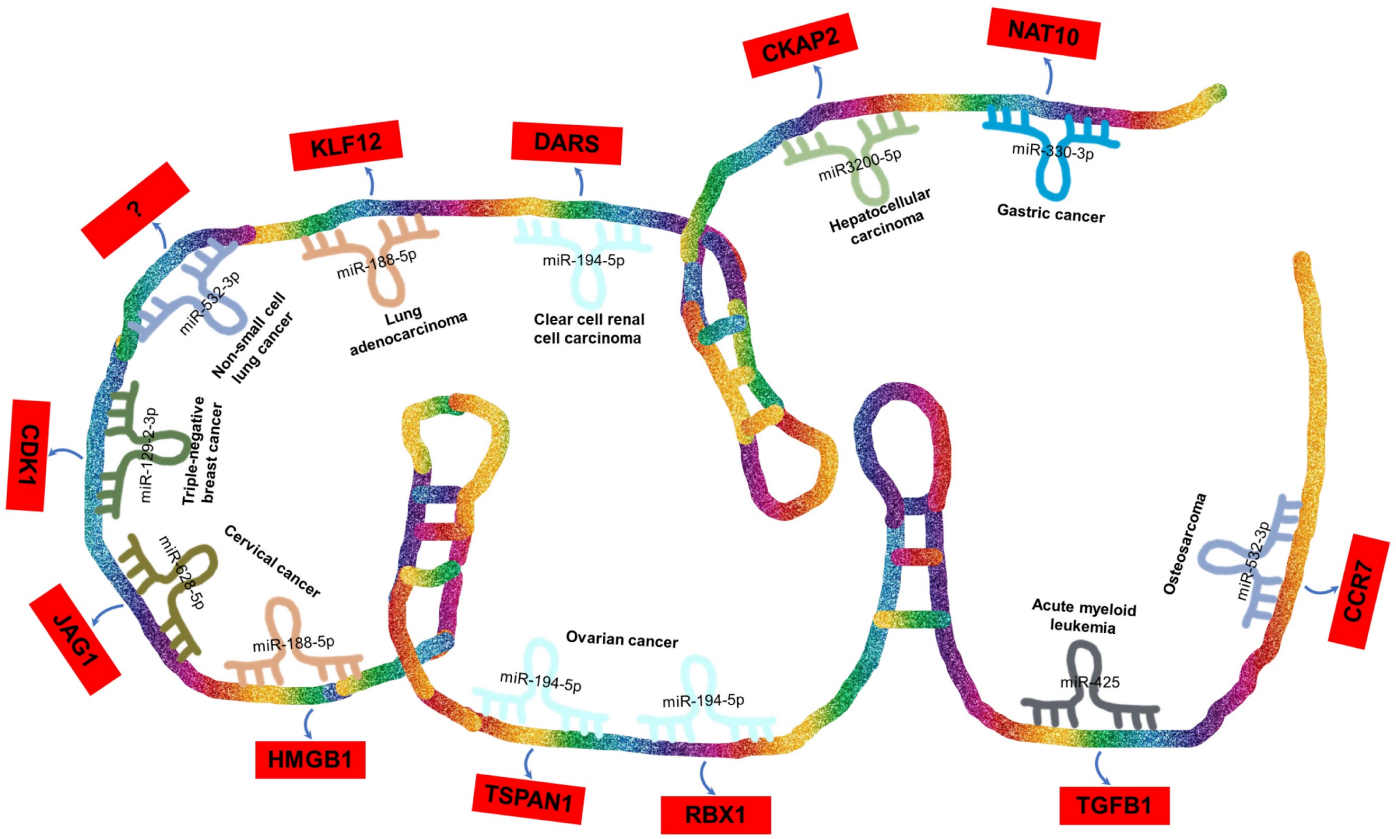
ceRNA Networks Showcasing lncRNA DARS-AS1 Interactions Across Diverse Human Cancers. LncRNA DARS-AS1 upregulates target gene expression by sponging miRNAs, including miR-330-3p, miR-3200-5p, miR-194-5p, miR-188-5p, miR-532-3p, and miR-129-2. This modulates the progression of various tumors, including gastric, liver, kidney, lung, breast, cervical, and ovarian cancers, alongside acute myeloid leukemia and osteosarcoma. **Abbreviations:** GC: Gastric cancer; HCC: Hepatocellular carcinoma; ccRCC: Clear cell renal cell carcinoma; LUAD: Lung adenocarcinoma; NSCLC: Non-small cell lung cancer; TNBC: Triple-negative breast cancer; CC: Cervical cancer; OC: Ovarian cancer; AML: Acute myeloid leukemia; OSA: Osteosarcoma; NAT10: N-Acetyltransferase 10; CKAP2: Cytoskeleton Associated Protein 2; DARS: Aspartyl-tRNA synthetase; KLF12:KLF Transcription Factor 12; CDK1:Cyclin Dependent Kinase 1; JAG1:Jagged Canonical Notch Ligand 1; HMGB1:High mobility group box 1; TSPAN1: Traspanin 1; RBX1:Ring-Box 1; TGFB1:Transforming growth factor beta 1; CCR7: C-C chemokine receptor type 7.

In the case of DARS-AS1, its ceRNA network encompasses eight miRNAs across nine different types of cancers, as illustrated in **Figure 4**. These miRNAs include miR-330-3p in gastric cancer 51, miR-3200-5p in hepatocellular carcinoma 35, miR-194-5p in clear cell renal cell carcinoma 36 and ovarian cancer 38, 48, miR-188-5p in lung adenocarcinoma 42 and cervical cancer 49, miR-532-3p in non-small cell lung cancer 39 and osteosarcoma 45, miR-129-2-3p in triple-negative breast cancer 40, miR-628-5p in cervical cancer 33, and miR-425 in childhood acute myeloid leukemia 34.

BBOX1-AS1 exerts its regulatory influence across various types of tumors by competitively binding to a diverse range of miRNAs. Furthermore, it contributes to the progression of distinct tumor types by influencing common miRNAs. Intriguingly, in the context of a single tumor, BBOX1-AS1 can engage with multiple miRNAs to impact tumor development. For instance, in cervical cancer 33, 49, BBOX1-AS1 engages in two distinct ceRNA mechanisms, thereby promoting both the proliferation and apoptosis of cervical cancer cells. These mechanisms involve the targeting of miR-628-5p/JAG1 33 and miR-188-5p/HMGB1 49.

### LncRNA DARS-AS1 as Functional Modules in Tumors

DARS-AS1 is a versatile regulator of gene expression, operating through ceRNA networks, as described above. However, it also exerts its influence in gene regulation by functioning as a modular scaffold, protein decoy, and molecular mediator impacting downstream targets.

When functioning as a molecular mediator, in triple-negative breast cancer 41, DARS-AS1 overexpression significantly upregulated the levels of TGF-β, p-Smad3, ATG5, and the conversion from LC3-I to LC3-II. Conversely, silencing DARS-AS1 reversed these effects. Silencing DARS-AS1 enhanced the sensitivity of triple-negative breast cancer (TNBC) cells to doxorubicin by suppressing autophagy induced by the TGF-β/Smad3 signaling pathway, thereby strengthening the synergistic antitumor effects. In cervical cancer 37, ATP1B2 was identified as a target mRNA of DARS-AS1, and it showed a negative correlation with DARS-AS1 expression. DARS-AS1/ATP1B2 partially regulated malignant behaviors through the cGMP-PKG signaling pathway.

When serving as a modular scaffold, in cervical cancer 43, DARS-AS1 enhanced DARS mRNA stability and translation by recruiting METTL3 and METTL14. Moreover, DARS-AS1 positively regulated IGF2BP3 expression by stabilizing IGF2BP3 mRNA. In glioblastoma 47, DARS1-AS1 interacted with YBX1 to promote the binding and stability of target mRNA. This established a mixed transcriptional/post-transcriptional feed-forward loop, enhancing the expression of key regulators of the G1-S transition, including E2F1 and CCND1. DARS1-AS1/YBX1 also increased the stability of FOXM1 mRNA, a master transcription factor regulating GSC self-renewal and DSB repair.

When acting as a protein decoy, in colorectal cancer 46, DARS-AS1 directly bound to PACT. This interaction inhibited the association between PACT and PKR, preventing the phosphorylation of the PKR downstream substrate eIF2α, ultimately inhibiting apoptotic cell death. In breast cancer and HCC 46, DARS-AS1 promoted cancer cell proliferation and inhibited apoptosis by inhibiting the function of PACT. In myeloma 44, DARS-AS1 exerts its function by binding RNA-binding motif protein 39 (RBM39), which impedes the interaction between RBM39 and its E3 ubiquitin ligase RNF147 and prevents RBM39 from degradation.

The intricate interplay of ceRNA networks and DARS-AS1's interactions with both mRNA and protein constituents endow it with a multifaceted role as a gene expression regulator. The perturbation of DARS-AS1 in cancer underscores its significance as a plausible therapeutic target, underscoring the urgency for in-depth exploration of its exact functionalities and molecular associations.

### Signaling pathways influenced by lncRNA DARS-AS1

**Figure 5:**
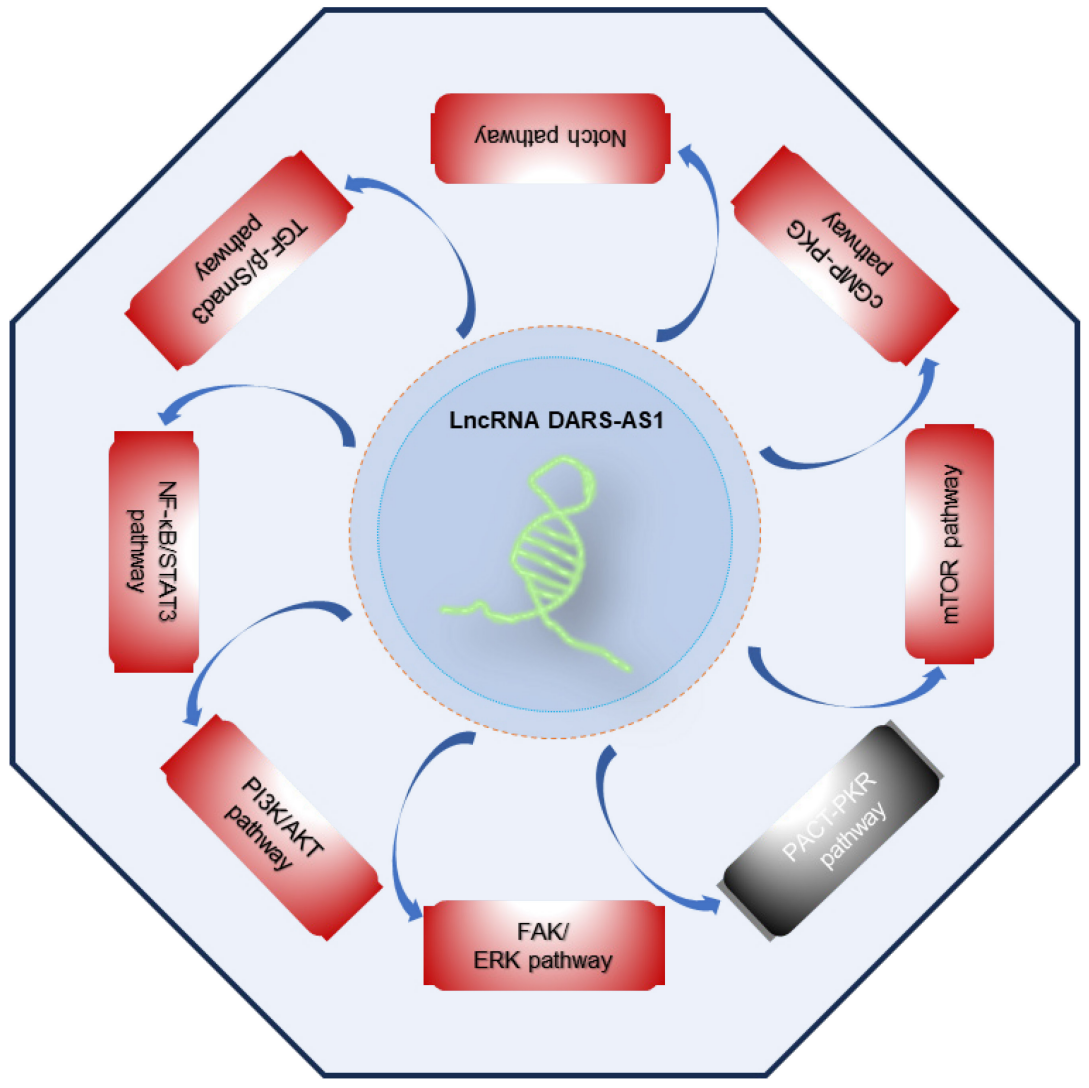
Signaling pathways regulated by lncRNA DARS-AS1. LncRNA DARS-AS1 promotes the tumor progression by activating multiple pathways, including FAK/ERK, PI3K/AKT, NF-κB/STAT3, TGF-β/Smad3, Notch, cGMP-PKG, and mTOR, as well as inactivating the PACT-PKR pathway. The red box indicates activation, the gray box indicates inactivation. **Abbreviations:** FAK/ERK: Focal adhesion kinase/Extracellular signal-regulated kinase; PI3K/AKT: Phosphoinositide 3-Kinase/Serine-threonine protein kinase; NF-κB/STAT3: Nuclear factor-kappa B/Signal transducer and activator of transcription 3; TGF-β/Smad3: Transforming growth factor beta/Smad family member 3; cGMP-PKG:Cyclic guanosine monophosphate/Protein kinase G; mTOR: Mammalian target of rapamycin; PACT-PKR: Protein activator of the interferon-induced protein kinase/Protein kinase R.

Accumulating scientific findings support the pivotal role of lncRNAs in orchestrating various signaling pathways 63-67, offering fresh perspectives for the development of targeted therapies. Presently, DARS-AS1 has been unequivocally established as an important participant in the regulation of multiple cancer-related signaling pathways, as outlined in **Figure 5**. These pathways encompass the FAK/ERK, PI3K/AKT, NF-κB/STAT3, TGF-β/Smad3, Notch, cGMP-PKG, mTOR, and PACT-PKR pathways. The involvement of DARS-AS1 in these intricate signaling networks implies its broader influence on the behavior of cancer cells and their responses to therapy.

In hepatocellular carcinoma 35, DARS-AS1 up-regulates CKAP2 by binding to miR-3200-5p, thereby activating the FAK-ERK pathway and promoting HCC proliferation and metastasis, and DARS-AS1 is also reported to promote HCC cell proliferation and inhibits apoptosis through inhibiting the function of PACT 46. In lung adenocarcinoma 42, it was found that DARS-AS1 significantly enhances the malignant properties of LUAD cells. This effect was achieved through the activation of the PI3K/AKT pathway, triggering the EMT process, and the up-regulation of Cyclin D1 and Bcl-2 proteins, both recognized contributors to cell growth and survival. In breast cancer, DARS-AS1 promote the TNBC tumorigenesis by activation of the NF-κB/STAT3 signaling pathway 40, and increased the resistance of TNBC cells to doxorubicin by promote TGF-β/Smad3 signaling pathway-induced autophagy 41. In addition, DARS-AS1 inhibits breast cancer cell proliferation, which is, at least partially, through repressing PACT-mediated PKR activation 46. In cervical cancer, DARS-AS1 exhibits pro-tumorigenic effects by activating the Notch pathway 33, and cGMP-PKG pathway 37, exacerbating the tumorigenesis of cervical cancer. In myeloma, Tong et al. 44 revealed that hypoxia-induced lncRNA DARS-AS1 upregulates RBM39 protein expression via the ubiquitin-proteasome pathway, and further promotes mTOR signaling pathway to promote myeloma malignancy. In colorectal cancer 46, lncRNA DARS-AS1 is directly involved in the inhibition of the PACT-PKR pathway and promotes the proliferation and inhibit cancer cell apoptosis, and promote tumor growth *in vivo*.

### Treatment resistance mediated by lncRNA DARS-AS1

Treatment resistance is a significant concern in cancer 68-71, and there is abundant evidence that lncRNAs play a role in regulating the sensitivity of tumor cells to drug and radiotherapy 72-77, thereby affecting tumor recurrence and metastasis.

Doxorubicin is the primary chemotherapy drug utilized to enhance the survival of triple-negative breast cancer patients 78-80. However, it often leads to strong drug resistance during its usage. Liu et al. 41 reported that silencing DARS-AS1 decreases doxorubicin resistance by suppressing autophagy via inhibition of the TGF-β/Smad3 signaling pathway, and combination of DARS-AS1 siRNA and DOX significantly inhibited tumorigenesis and growth of TNBC cells, which indicated that combination of DARS-AS1 siRNA and doxorubicin can be used as new therapeutic agents for TNBC.

In the context of myeloma 44, lncRNA DARS-AS1 is directly upregulated by hypoxia inducible factor-1. And overexpression of DARS-AS1 resulted in a decreased responsiveness of myeloma cells to bortezomib. Additionally, Zheng et al. 47 investigated the impact of BBOX1-AS1 on glioblastoma tumorigenesis/radioresistance by multiomics analyses, and revealed that DARS1-AS1 depletion impaired the homologous recombination (HR)-mediated double-strand break (DSB) repair and enhanced the radiosensitivity of glioblastoma cells.

Overall, exploring the molecular mechanisms of the DARS1-AS1-associated regulatory axis in drug and radiotherapy resistance could provide valuable insights for the development of targeted therapies and improved clinical outcomes for cancer patients.

## Future perspectives

**Figure 6:**
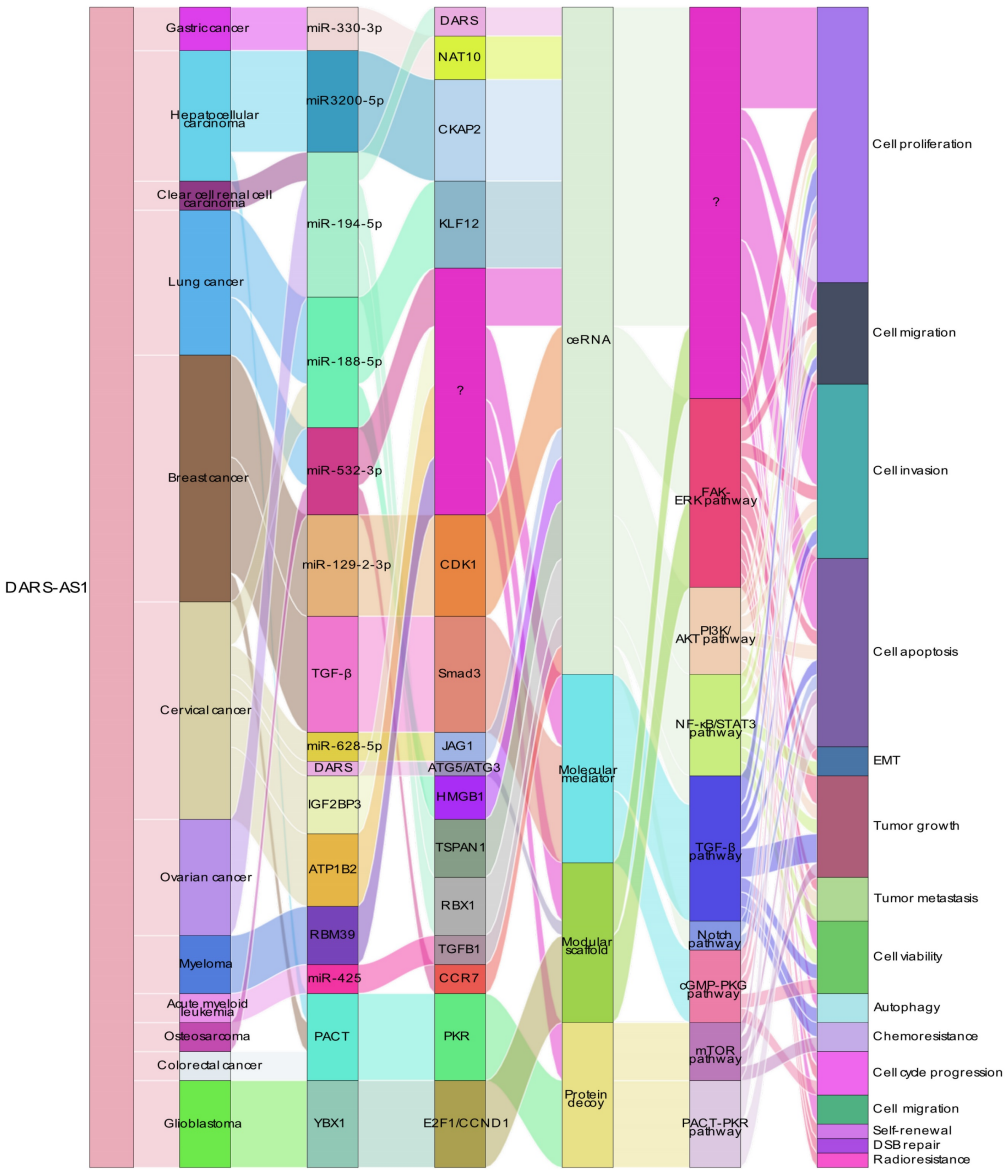
Summary of the molecular mechanisms of lncRNA DARS-AS1 as a central regulator of cellular processes in cancer. This figure provides an overview of how lncRNA DARS-AS1 serves as a central regulator of cellular processes in cancer. It influences gene expression through ceRNA networks and functions as a versatile entity. DARS-AS1 acts as a modular scaffold, protein decoy, and molecular mediator, thereby impacting downstream targets in gene regulation.

In recent years, DARS1-AS1, as an emerging oncogenic lncRNA, has been consistently found to be upregulated in various cancer types. Notably, DARS1-AS1 demonstrates significant clinical relevance in prognostic predictions. Studies have revealed that DARS1-AS1 plays a regulatory role in tumor development by influencing key molecules and genes involved in critical tumor-related biological processes, as shown in **Figure 6.** These findings highlight the potential therapeutic implications of targeting DARS1-AS1 in cancer treatment.

In terms of mechanisms, DARS-AS1 plays a crucial role as a multifaceted functional module, encompassing activities such as ceRNA, modular scaffold, protein decoy, and molecular mediator functions. It intricately influences gene expression and modulates signaling pathways, thereby regulating biological processes associated with tumorigenesis and progression. As a ceRNA, lncRNA DARS-AS1 binds to microRNA, leading to the upregulation of target genes such as NAT10, DARS, CDK1, JAG1, and HMGB1, and thus exhibits a broader regulatory effect on the development of different tumors. Notably, lncRNA DARS-AS1 is intricately involved in many signaling pathways, such as FAK/ERK, PI3K/AKT, NF-κB/STAT3, TGF-β/Smad3, Notch, cGMP-PKG, mTOR. Among these pathways, lncRNA DARS-AS1 plays an activating role, affecting key processes in cancer initiation and progression. By engaging with these signaling pathways, lncRNA DARS-AS1 plays a central role in shaping the dynamics of cancer cells, promoting their survival and enhancing their invasive and metastatic potential. And lncRNA DARS-AS1 inactivates PACT-PKR pathway, promotes proliferation and inhibits apoptotic cell death in multiple cancer cells. Remarkably, diminished levels of DARS1-AS1 expression demonstrated heightened susceptibility to both drug and radiation therapies, suggesting a potential avenue for augmenting the efficacy of current cancer treatment modalities. Overall, the intricate array of functions attributed to DARS1-AS1, encompassing its role as a ceRNA, functional modules, and its influence on pivotal signaling pathways, positions it as a promising therapeutic target for pioneering cancer interventions designed to combat metastasis and enhance treatment responses.

Despite the progress made, our understanding of DARS1-AS1 remains incomplete. Further studies are needed to assess DARS1-AS1 expression in hematopoietic cancers and to determine its impact on tumor progression and prognosis in larger study populations of different tumor types. In addition, no studies have explored the diagnostic potential of DARS1-AS1. It is imperative to evaluate the diagnostic value of DARS1-AS1 for cancer. In particular, the clinical value of detection of DARS1-AS1 in liquid tissue in the early diagnosis of tumors deserves further study. Furthermore, it is essential to acquire a more profound understanding of the specific regulatory mechanisms of DARS1-AS1 in various tumor types. DARS1-AS1 might be intricately connected to additional signaling pathways and could possess a broader ceRNA network. *In vitro* and *in vivo* studies are needed to elucidate the mechanisms through which DARS1-AS1 contributes to therapy resistance across diverse tumor types.

## Conclusion

In a nutshell, DARS-AS1 is an oncogenic lncRNA consistently overexpressed in various cancers, strongly linked to poor patient outcomes. Research in lab and live settings reveals its cancer-promoting role in tumor processes through ceRNA networks and signaling pathways. DARS-AS1 holds promise as a cancer biomarker and therapeutic target, but more research and clinical validation are needed to fully understand its mechanisms and potential applications in cancer management.

## Author contributions

HLL and YW designed and supervised the study. JS and KJX wrote the first draft. HLL and YW edited the manuscript. JS, KJX, HLL, and YW collected reference materials and produced tables and graphical visualizations. All authors read and approved the final manuscript.

## Figures and Tables

**Table 1:** Correlations between the expression levels of lncRNA DARS-AS1 in tumor tissues, clinical characteristics, and prognostic outcomes.

Tumor type	Expression in tumor tissues	Clinical features	Prognosis	Methods for survival analysis	Indicator for poor survival	Ref.
Gastric cancer	Up-regulated	T category, N category, TNM stage	OS	K-M plot; Multivariate analysis	High expression	51
Hepatocellular carcinoma	Up-regulated	TNM stage, Tumor size	OS	K-M plot; Multivariate analysis	High expression	35
Clear cell renal cell carcinoma	Up-regulated	Clinical stage	-	-	-	36
Lung adenocarcinoma	Up-regulated	-	OS	K-M plot	High expression	42
Non-small cell lung cancer	Up-regulated	-	-	-	-	39
Triple-negative breast cancer	Up-regulated	Clinical stage	OS	K-M plot	High expression	40
Cervical cancer	Up-regulated	-	-	-	-	33
Cervical cancer	Up-regulated	-	-	-	-	43
Cervical Cancer	Up-regulated	-	OS	K-M plot	High expression	49
Cervical Cancer	Up-regulated	-	-	-	-	50
Cervical cancer	Up-regulated	-	OS	K-M plot	High expression	37
Ovarian cancer	Up-regulated	-	-	-	-	38
Ovarian cancer	Up-regulated	-	-	-	-	48
Acute myeloid leukemia	Up-regulated	White blood cells, Hemoglobin, Platelet count	OS	K-M plot	High expression	34
Osteosarcoma	Up-regulated	-	-	-	-	45
Colorectal cancer	Up-regulated	-	-	-	-	46
Hepatocellular carcinoma	Up-regulated	-	-	-	-	46
UVM, KICH, KIRP, MESO, GBM, and LGG (TCGA datasets)	-	-	OS	K-M plot	High expression	46
BLCA, KIRC, PRAD, LUSC, UCEC, LUAD, LIHC, KIRP and COAD (TCGA datasets)	Up-regulated	-	-	-	-	46
Glioblastoma	Up-regulated	-	OS	K-M plot; Multivariate analysis	High expression	47

OS: Overall Survival, K-M plot: Kaplan-Meier plot, UVM: Uveal melanoma, KICH: Kidney chromophobe, KIRP: Kidney renal papillary cell carcinoma, MESO: Mesothelioma, GBM: Glioblastoma, LGG: lower grade glioma, BLCA: Bladder urothelial carcinoma, PRAD: Prostate adenocarcinoma, LUSC: Lung squamous cell carcinoma, UCEC: Uterine corpus endometrial carcinoma, LUAD: Lung adenocarcinoma, LIHC: Liver hepatocellular carcinoma, COAD: Colon adenocarcinoma, TCGA: The Cancer Genome Atlas. "-": Indicates missing or not applicable data.

**Table 2:** Functions and regulatory mechanisms of lncRNA DARS-AS1 in different cancers.

Cancer Type	Utilized Cell Lines	Expression Levels in Cancer Cell Lines	Subcellular localization	Animal models	Regulatory Pathways	Functional Role of LncRNA DARS-AS1	Effects *in vitro*	Effects *in vivo*	Signaling pathway	Resistance	Ref.
Gastric cancer	AGS, KATO-III, HGC-27, NCI-N87, MKN45, SNU-1, GES-1	Up- regulated	-	-	DARS-AS1/miR-330-3p/NAT10	ceRNA	Proliferation, migration, invasion	-	-	-	51
Hepatocellular carcinoma	THLE-3, Huh-7, HCCLM3, HLE, MHCC97, HCCLM6	Up- regulated	-	BALB/c mice (male, 6-8 weeks old)	DARS-AS1/miR3200-5p/CKAP2	ceRNA	Proliferation, apoptosis, migration, invasion, EMT	Tumor growth and metastasis	FAK-ERK pathway	-	35
Clear cell renal cell carcinoma	HK-2, ClearCa1, HH332, Caki-1, KMRC-2, KN-41	Up- regulated	Mainly in cytoplasm	-	DARS-AS1/miR-194-5p/DARS	ceRNA	Proliferation, apoptosis	-	-	-	36
Lung adenocarcinoma	NCI-H23, A549, HCC827, PC-9, C422L, HBE	Up- regulated	-	Orthotopic xenografts and subcutaneous tumor transplantation	DARS-AS1/miR-188-5p/KLF12	ceRNA	Proliferation, invasion, migration, apoptosis	Tumor growth and metastasis	PI3K/AKT pathway	-	42
Non-small cell lung cancer	SPca1, H1299, Pc-9, H358, 16HBe	Up- regulated	-	-	DARS-AS1/miR-532-3p	ceRNA	Cell viability, proliferation, invasion, migration	-	-	-	39
Triple-negative breast cancer	MDA-MB-231, MDA-MB-468, BT549,MCF-10A	Up- regulated	-	Xenograft model (Female BALB/c nude mice)	DARS-AS1/miR-129-2-3p/CDK1	ceRNA	Cell viability, proliferation, migration, invasion, EMT	Tumor growth and metastasis	NF-κB/STAT3 pathway	-	40
Triple-negative breast cancer	MDA-MB-231, MDA-MB-468, BT549, MDA-MB-231/ADR, MCF-10A	-	-	Orthotopic models (6 weeks old female BALB/c nude mice)	DARS-AS1/TGF-β/Smad3	Molecular mediator	Proliferation, migration, invasion, apoptosis, autophagy, cell viability	Tumor growth	TGF-β pathway	Chemoresistance (Doxorubicin)	41
Cervical cancer	HeLa, C33A, MS751, SiHa, ME-180, CaSki, Ect1/E6E7	Up- regulated	Mainly in cytoplasm	-	DARS-AS1/miR-628-5p/JAG1	ceRNA	Proliferation cell apoptosis	-	Notch pathway	-	33
Cervical cancer	SiHa, CaSki, C33A, DoTc24510, HeLa, End1/E6E7	Up- regulated	Both in nucleus and cytoplasm	-	HIF1α/DARS-AS1/ DARS/ATG5/ATG3	Modular scaffold	Autophagy	-	-	-	43
Cervical Cancer	SiHa, HeLa	-	-	-	DARS-AS1/miR-188-5p/HMGB1	ceRNA	Proliferation, apoptosis, invasion	-	-	-	49
Cervical Cancer	SiHa, Hela	-	Mainly in cytoplasm	subcutaneous xenograft model (BALB/c nude mice, 4-6-weeks old)	DARS-AS1/ IGF2BP3	Modular scaffold	Proliferation, apoptosis, invasion, cell cycle progression	Tumor growth	-	-	50
Cervical cancer	SiHa, CaSki, C-33A, Ect1/ E6E7	Up- regulated	-	-	DARS-AS1/ ATP1B2	Molecular mediator	Cell viability, proliferation, invasion, migration, cell cycle progression	-	cGMP-PKG pathway	-	37
Ovarian cancer	Caov-3, A2780, SKOV3, CoC1, IOSE80	Up- regulated	-	-	DARS-AS1/miR-194-5p/TSPAN1 /ITGA2	ceRNA	Cell viability, migration, invasion, apoptosis	-	-	-	38
Ovarian cancer	A2780, HeyA8, SKOV3	Up- regulated	-	-	DARS-AS1/miR-194-5p/RBX1 /TP53	ceRNA	Proliferation, apoptosis, invasion, migration	-	-	-	48
Myeloma	RPMI 8226, LP-1, U266, H929	Up- regulated	-	NOD-SCID mice	HIF-1/DARS-AS1/ RBM39	Protein decoy	Proliferation, apoptosis	Tumor growth	mTOR pathway	Chemoresistance (Bortezomib)	44
Acute myeloid leukemia	HS-5, BF-24, MV4-11, U937, HL-60	Up- regulated	In the cytoplasm	Six-week-old male NOD/SCID mice	DARS-AS1/miR-425/TGFB1	ceRNA	Proliferation	Tumor growth	TGF-β pathway	/	34
Osteosarcoma	U2OS, SOSP-9607, Saos-2, MG-63, hFOB	Up- regulated	-	-	DARS-AS1/miR-532-3p/CCR7	ceRNA	Proliferation, invasion	-	-	-	45
Colorectal cancer	SW620 HCT116	-	Mainly in cytoplasm	Xenograft mouse models	DARS-AS1/ PACT/ PKR	Protein decoy	Cell proliferation, apoptosis	Tumor growth	PACT-PKR pathway	-	46
Breast cancer	MBA-MD-231	-	-	-	DARS-AS1/ PACT / PKR	Protein decoy	Cell proliferation, apoptosis	-	PACT-PKR pathway	-	46
Hepatocellular carcinoma	HepG2	-	-	-	DARS-AS1/ PACT / PKR	Protein decoy	Cell proliferation, apoptosis	-	PACT-PKR pathway	-	46
Glioblastoma	U251, U87, LN229, GSC11, GSC17, GSC20, GSC262, GSC272, GSC295, Hs683, SW1783, NHAs, ReNcell	Up- regulated	Mainly in cytoplasm	Orthotopic glioblastoma models (athymic nude mice,6-8-week-old female)	DARS1-AS1/YBX1/E2F1/CCND1	Modular scaffold	Proliferation, self-renewal, DSB repair, cell cycle progression	Tumor growth, Survival	-	Radioresistance	47

ceRNA: Competing endogenous RNA, DSB: Double-strand break, "-": Indicates missing or not applicable data.
